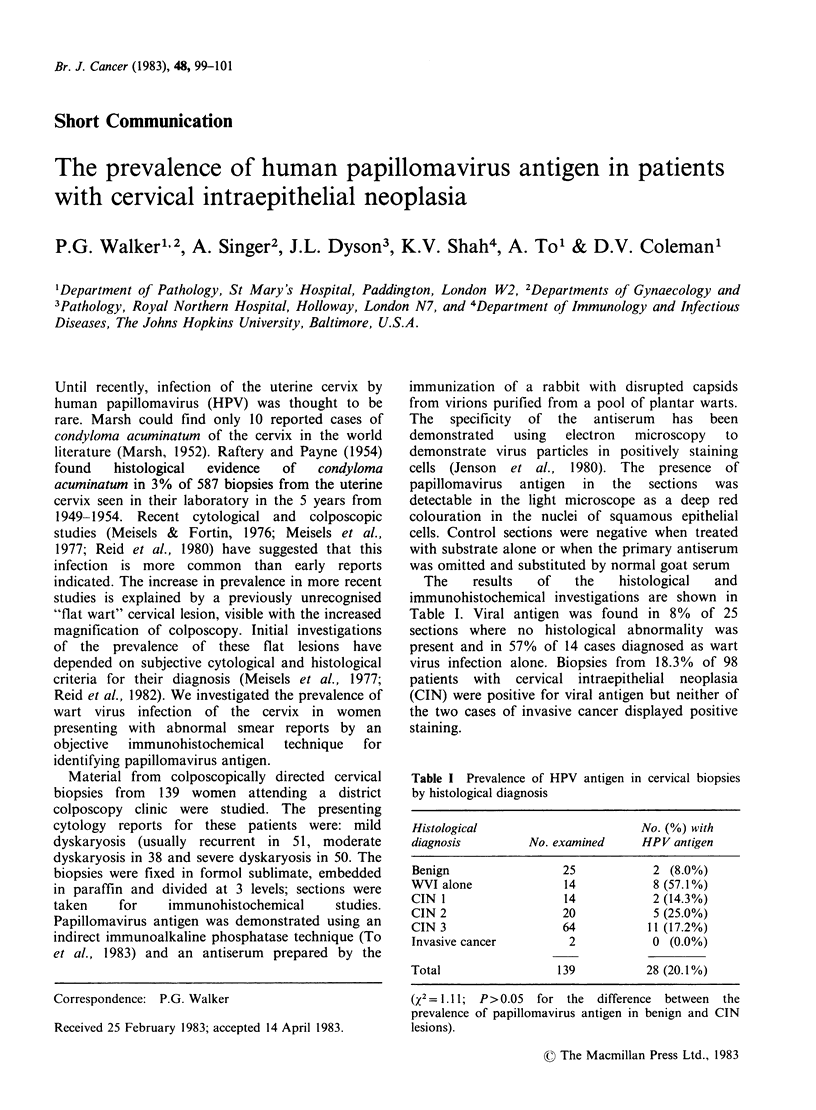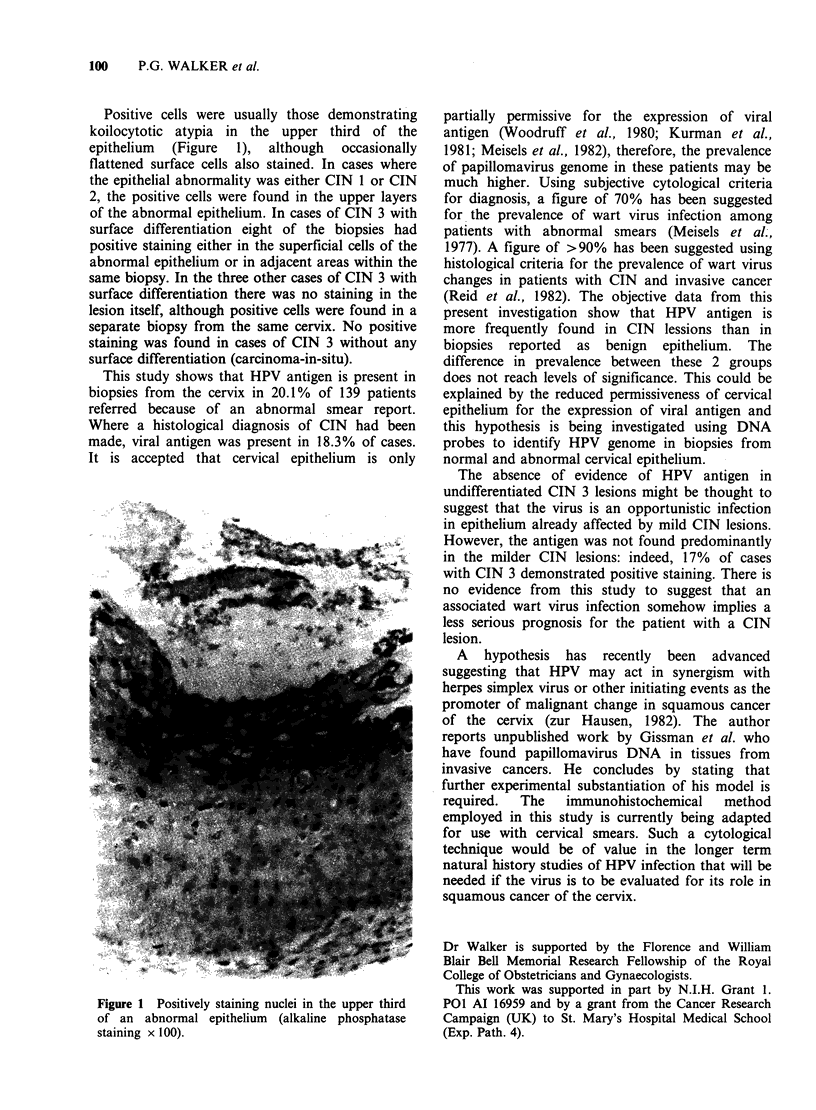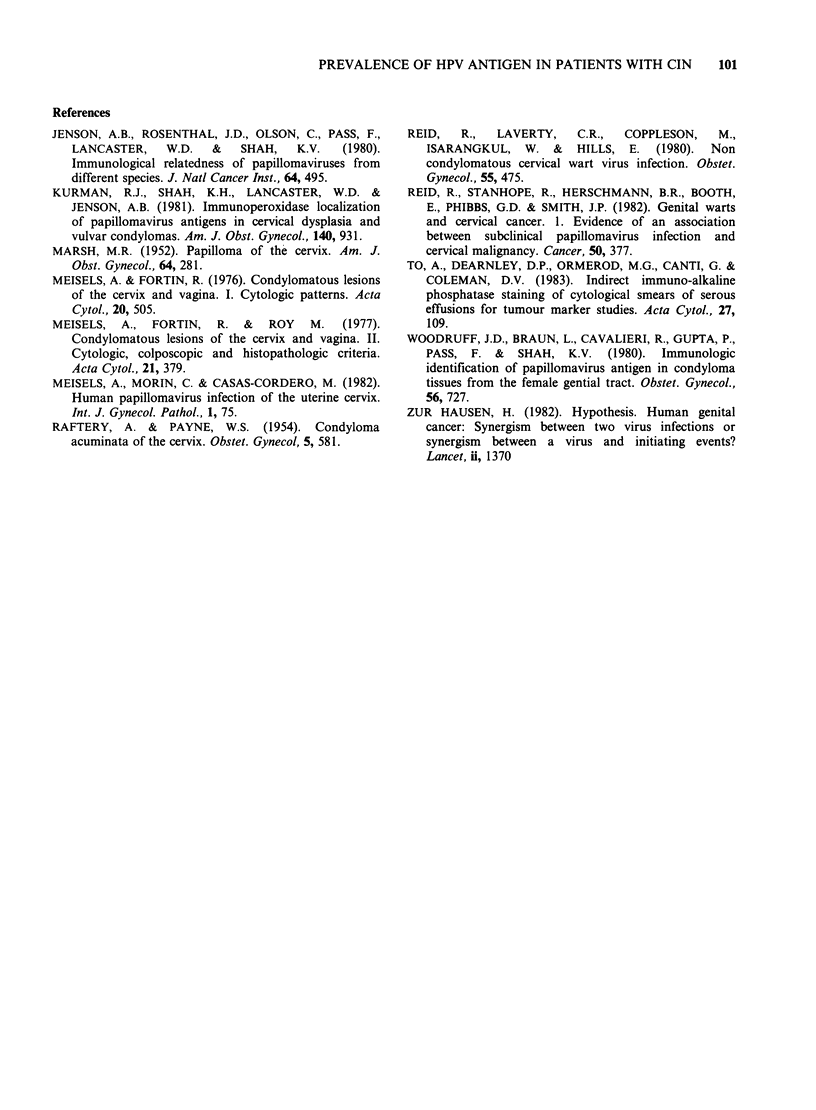# The prevalence of human papillomavirus antigen in patients with cervical intraepithelial neoplasia.

**DOI:** 10.1038/bjc.1983.163

**Published:** 1983-07

**Authors:** P. G. Walker, A. Singer, J. L. Dyson, K. V. Shah, A. To, D. V. Coleman

## Abstract

**Images:**


Br. J. Cancer (1983), 48, 99–101

### Short Communication

### The prevalence of human papillomavirus antigen in patients with cervical intraepithelial neoplasia

P.G. Walker[1,2], A. Singer[2], J.L. Dyson[3], K.V. Shah[4], A. To[1] & D.V. Coleman[1]

[1]*Department of Pathology, St Mary's Hospital, Paddington, London W2,* [2]*Departments of Gynaecology and* [3]*Pathology, Royal Northern Hospital, Holloway, London N7, and* [4]*Department of Immunology and Infectious Diseases, The Johns Hopkins University, Baltimore, U.S.A.*

Until recently, infection of the uterine cervix by human papillomavirus (HPV) was thought to be rare. Marsh could find only 10 reported cases of *condyloma acuminatum* of the cervix in the world literature (Marsh, 1952). Raftery and Payne (1954) found histological evidence of *condyloma acuminatum* in 3% of 587 biopsies from the uterine cervix seen in their laboratory in the 5 years from 1949–1954. Recent cytological and colposcopic studies (Meisels & Fortin, 1976; Meisels *et al.*, 1977; Reid *et al.*, 1980) have suggested that this infection is more common than early reports indicated. The increase in prevalence in more recent studies is explained by a previously unrecognised "flat wart" cervical lesion, visible with the increased magnification of colposcopy. Initial investigations of the prevalence of these flat lesions have depended on subjective cytological and histological criteria for their diagnosis (Meisels *et al.*, 1977; Reid *et al.*, 1982). We investigated the prevalence of wart virus infection of the cervix in women presenting with abnormal smear reports by an objective immunohistochemical technique for identifying papillomavirus antigen.

Material from colposcopically directed cervical biopsies from 139 women attending a district colposcopy clinic were studied. The presenting cytology reports for these patients were: mild dyskaryosis (usually recurrent in 51, moderate dyskaryosis in 38 and severe dyskaryosis in 50. The biopsies were fixed in formol sublimate, embedded in paraffin and divided at 3 levels; sections were taken for immunohistochemical studies. Papillomavirus antigen was demonstrated using an indirect immunoalkaline phosphatase technique (To *et al.*, 1983) and an antiserum prepared by the immunization of a rabbit with disrupted capsids from virions purified from a pool of plantar warts. The specificity of the antiserum has been demonstrated using electron microscopy to demonstrate virus particles in positively staining cells (Jenson *et al.*, 1980). The presence of papillomavirus antigen in the sections was detectable in the light microscope as a deep red colouration in the nuclei of squamous epithelial cells. Control sections were negative when treated with substrate alone or when the primary antiserum was omitted and substituted by normal goat serum

The results of the histological and immunohistochemical investigations are shown in Table I. Viral antigen was found in 8% of 25 sections where no histological abnormality was present and in 57% of 14 cases diagnosed as wart virus infection alone. Biopsies from 18.3% of 98 patients with cervical intraepithelial neoplasia (CIN) were positive for viral antigen but neither of the two cases of invasive cancer displayed positive staining.

**Table I** Prevalence of HPV antigen in cervical biopsies by histological diagnosis

| *Histological diagnosis* | *No. examined* | *No. (%) with HPV antigen* |
|---|---|---|
| Benign | 25 | 2 (8.0%) |
| WVI alone | 14 | 8 (57.1%) |
| CIN 1 | 14 | 2 (14.3%) |
| CIN 2 | 20 | 5 (25.0%) |
| CIN 3 | 64 | 11 (17.2%) |
| Invasive cancer | 2 | 0 (0.0%) |
| Total | 139 | 28 (20.1%) |

($\chi^2 = 1.11$; $P > 0.05$ for the difference between the prevalence of papillomavirus antigen in benign and CIN lesions).

Correspondence: P.G. Walker

Received 25 February 1983; accepted 14 April 1983.

© The Macmillan Press Ltd., 1983

Positive cells were usually those demonstrating koilocytotic atypia in the upper third of the epithelium (Figure 1), although occasionally flattened surface cells also stained. In cases where the epithelial abnormality was either CIN 1 or CIN 2, the positive cells were found in the upper layers of the abnormal epithelium. In cases of CIN 3 with surface differentiation eight of the biopsies had positive staining either in the superficial cells of the abnormal epithelium or in adjacent areas within the same biopsy. In the three other cases of CIN 3 with surface differentiation there was no staining in the lesion itself, although positive cells were found in a separate biopsy from the same cervix. No positive staining was found in cases of CIN 3 without any surface differentiation (carcinoma-in-situ).

This study shows that HPV antigen is present in biopsies from the cervix in 20.1% of 139 patients referred because of an abnormal smear report. Where a histological diagnosis of CIN had been made, viral antigen was present in 18.3% of cases. It is accepted that cervical epithelium is only partially permissive for the expression of viral antigen (Woodruff *et al.*, 1980; Kurman *et al.*, 1981; Meisels *et al.*, 1982), therefore, the prevalence of papillomavirus genome in these patients may be much higher. Using subjective cytological criteria for diagnosis, a figure of 70% has been suggested for the prevalence of wart virus infection among patients with abnormal smears (Meisels *et al.*, 1977). A figure of >90% has been suggested using histological criteria for the prevalence of wart virus changes in patients with CIN and invasive cancer (Reid *et al.*, 1982). The objective data from this present investigation show that HPV antigen is more frequently found in CIN lessions than in biopsies reported as benign epithelium. The difference in prevalence between these 2 groups does not reach levels of significance. This could be explained by the reduced permissiveness of cervical epithelium for the expression of viral antigen and this hypothesis is being investigated using DNA probes to identify HPV genome in biopsies from normal and abnormal cervical epithelium.

**Figure 1** Positively staining nuclei in the upper third of an abnormal epithelium (alkaline phosphatase staining ×100).

The absence of evidence of HPV antigen in undifferentiated CIN 3 lesions might be thought to suggest that the virus is an opportunistic infection in epithelium already affected by mild CIN lesions. However, the antigen was not found predominantly in the milder CIN lesions: indeed, 17% of cases with CIN 3 demonstrated positive staining. There is no evidence from this study to suggest that an associated wart virus infection somehow implies a less serious prognosis for the patient with a CIN lesion.

A hypothesis has recently been advanced suggesting that HPV may act in synergism with herpes simplex virus or other initiating events as the promoter of malignant change in squamous cancer of the cervix (zur Hausen, 1982). The author reports unpublished work by Gissman *et al.* who have found papillomavirus DNA in tissues from invasive cancers. He concludes by stating that further experimental substantiation of his model is required. The immunohistochemical method employed in this study is currently being adapted for use with cervical smears. Such a cytological technique would be of value in the longer term natural history studies of HPV infection that will be needed if the virus is to be evaluated for its role in squamous cancer of the cervix.

Dr Walker is supported by the Florence and William Blair Bell Memorial Research Fellowship of the Royal College of Obstetricians and Gynaecologists.

This work was supported in part by N.I.H. Grant 1. PO1 AI 16959 and by a grant from the Cancer Research Campaign (UK) to St. Mary's Hospital Medical School (Exp. Path. 4).

### References

JENSON, A.B., ROSENTHAL, J.D., OLSON, C., PASS, F., LANCASTER, W.D. & SHAH, K.V. (1980). Immunological relatedness of papillomaviruses from different species. *J. Natl Cancer Inst.*, **64**, 495.

KURMAN, R.J., SHAH, K.H., LANCASTER, W.D. & JENSON, A.B. (1981). Immunoperoxidase localization of papillomavirus antigens in cervical dysplasia and vulvar condylomas. *Am. J. Obst. Gynecol.*, **140**, 931.

MARSH, M.R. (1952). Papilloma of the cervix. *Am. J. Obst. Gynecol.*, **64**, 281.

MEISELS, A. & FORTIN, R. (1976). Condylomatous lesions of the cervix and vagina. I. Cytologic patterns. *Acta Cytol.*, **20**, 505.

MEISELS, A., FORTIN, R. & ROY M. (1977). Condylomatous lesions of the cervix and vagina. II. Cytologic, colposcopic and histopathologic criteria. *Acta Cytol.*, **21**, 379.

MEISELS, A., MORIN, C. & CASAS-CORDERO, M. (1982). Human papillomavirus infection of the uterine cervix. *Int. J. Gynecol. Pathol.*, **1**, 75.

RAFTERY, A. & PAYNE, W.S. (1954). Condyloma acuminata of the cervix. *Obstet. Gynecol*, **5**, 581.

REID, R., LAVERTY, C.R., COPPLESON, M., ISARANGKUL, W. & HILLS, E. (1980). Non condylomatous cervical wart virus infection. *Obstet. Gynecol.*, **55**, 475.

REID, R., STANHOPE, R., HERSCHMANN, B.R., BOOTH, E., PHIBBS, G.D. & SMITH, J.P. (1982). Genital warts and cervical cancer. 1. Evidence of an association between subclinical papillomavirus infection and cervical malignancy. *Cancer*, **50**, 377.

TO, A., DEARNLEY, D.P., ORMEROD, M.G., CANTI, G. & COLEMAN, D.V. (1983). Indirect immuno-alkaline phosphatase staining of cytological smears of serous effusions for tumour marker studies. *Acta Cytol.*, **27**, 109.

WOODRUFF, J.D., BRAUN, L., CAVALIERI, R., GUPTA, P., PASS, F. & SHAH, K.V. (1980). Immunologic identification of papillomavirus antigen in condyloma tissues from the female gential tract. *Obstet. Gynecol.*, **56**, 727.

ZUR HAUSEN, H. (1982). Hypothesis. Human genital cancer: Synergism between two virus infections or synergism between a virus and initiating events? *Lancet*, **ii**, 1370